# Redox Signaling of NADPH Oxidases Regulates Oxidative Stress Responses, Immunity and Aging

**DOI:** 10.3390/antiox7100130

**Published:** 2018-09-28

**Authors:** Collin Y. Ewald

**Affiliations:** Eidgenössische Technische Hochschule (ETH) Zürich, Department of Health Sciences and Technology, Institute of Translational Medicine, 8603 Schwerzenbach-Zürich, Switzerland; collin-ewald@ethz.ch

**Keywords:** NOX, sulfenylation, Nrf2, SKN-1, centenarians, extracellular matrix, longevity

## Abstract

An accumulating body of evidence suggests that transient or physiological reactive oxygen species (ROS) generated by nicotinamide adenine dinucleotide phosphate (NADPH) oxidases act as a redox signal to re-establish homeostasis. The capacity to re-establish homeostasis progressively declines during aging but is maintained in long-lived animals to promote healthy aging. In the model organism *Caenorhabditis elegans*, ROS generated by dual oxidases (Duox) are important for extracellular matrix integrity, pathogen defense, oxidative stress resistance, and longevity. The Duox enzymatic activity is tightly regulated and under cellular control. Developmental molting cycles, pathogen infections, toxins, mitochondrial-derived ROS, drugs, and small GTPases (e.g., RHO-1) can activate Duox (BLI-3) to generate ROS, whereas NADPH oxidase inhibitors and negative regulators, such as MEMO-1, can inhibit Duox from generating ROS. Three mechanisms-of-action have been discovered for the Duox/BLI-3-generated ROS: (1) enzymatic activity to catalyze crosslinking of free tyrosine ethyl ester in collagen bundles to stabilize extracellular matrices, (2) high ROS bursts/levels to kill pathogens, and (3) redox signaling activating downstream kinase cascades to transcription factors orchestrating oxidative stress and immunity responses to re-establish homeostasis. Although Duox function at the cell surface is well established, recent genetic and biochemical data also suggests a novel role for Duoxs at the endoplasmic reticulum membrane to control redox signaling. Evidence underlying these mechanisms initiated by ROS from NADPH oxidases, and their relevance for human aging, are discussed in this review. Appropriately controlling NADPH oxidase activity for local and physiological redox signaling to maintain cellular homeostasis might be a therapeutic strategy to promote healthy aging.

## 1. Introduction

How reactive oxygen species (ROS) influence the rate of aging is an unsolved mystery in biology. Chronic exposure to ROS accelerates the development of age-dependent diseases, such as Alzheimer’s disease, Parkinson’s disease, cancer, diabetes, cardiovascular diseases, and chronic inflammation. Thus, one could assume that using antioxidants to inactivate ROS would ameliorate accelerated development of these age-dependent diseases. However, in recent clinical trials, intake of antioxidants has been associated with a negative impact on human health [1,2,3]. Since ROS can cause molecular damage, organisms have evolved mechanisms that protect against abnormally high levels of ROS. Acute or low ROS exposure can initiate these protective mechanisms. In model organisms, this low or acute ROS exposure can increase lifespan of yeast [4,5,6], flies [7], nematodes [8,9,10,11,12,13], and rodents [14,15]. Low or acute ROS levels at the physiological level can act as a second messenger to alter cellular signaling, also known as redox signaling, and they are also important for adaptation against oxidative stress [16,17]. In cells, ROS can either originate as a by-product of mitochondrial oxidative phosphorylation [18] or be produced via enzymes, such as nicotinamide adenine dinucleotide phosphate (NADPH)-oxidases [19,20,21]. Since NADPH oxidases are under cellular control, the ROS generated by NADPH oxidases is ideal for localized activation of redox signaling.

**Figure 1:**
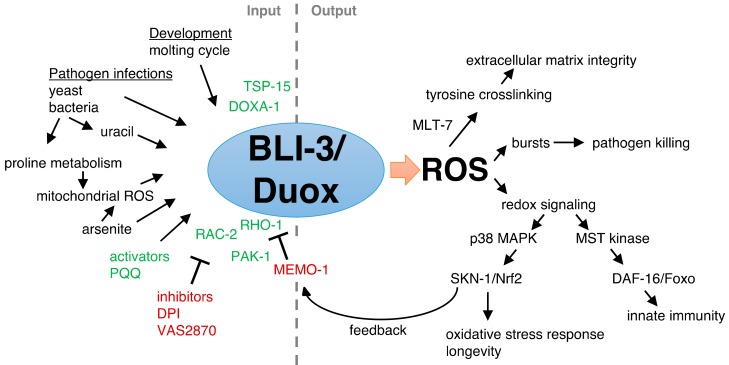
BLI-3/Duox activation and function. On the left side are the inputs that either directly or indirectly activate (arrows or green text) or inhibit (T-bar or red text) the BLI-3/Duox activity. On the right side are the outputs that are mediated by the BLI-3-generated ROS. Evidence and explanations are in the main text of this review. Pyrroloquinoline quinone (PQQ), diphenyleneiodonium (DPI).

In mammals, there are seven NADPH oxidases (NOX1-5 and Duox1-2). These NADPH oxidases are membrane-bound enzymes that generate superoxide, which is important for cellular signaling, development, apoptosis, protein modification, and protection against pathogens [19,20,21]. The roles of mammalian NADPH oxidases during aging are discussed below in the sections 9 and 10 of this review. Here, I focus more on the role of Dual oxidases (Duox) for redox signaling and aging. In addition to the superoxide-generating NADPH oxidase domain, Duoxs also have a peroxidase domain that converts the superoxide into hydrogen peroxide needed for the iodination of tyrosine in the thyroid hormone biosynthesis pathway in humans [22,23]. Mutations in Duox2 cause congenital hypothyroidism in humans [24]. Duoxs play important roles in tyrosine crosslinking, for instance, in sea urchins eggs [25], *Drosophila* wings [26], and in the *A. gambiae* gut [27]. Furthermore, Duox are expressed in the respiratory and gastrointestinal tract in mammals [28], where they potentially could function as a host defense mechanism against infections similar as in *Drosophila* [29,30] and zebrafish [31]. In *C. elegans*, Duox function is also important for tyrosine crosslinking [32], immunity [33], oxidative stress resistance [13,34], and healthy aging [13,35] (Figure 1). Here, I discuss how the *C. elegans* Duoxs become activated, and how the generated ROS acts in redox signaling to adapt to oxidative stress and re-establish cellular homeostasis.

## 2. Insights of Duox Functions from the Model Organism *C. elegans*

**Figure 2:**
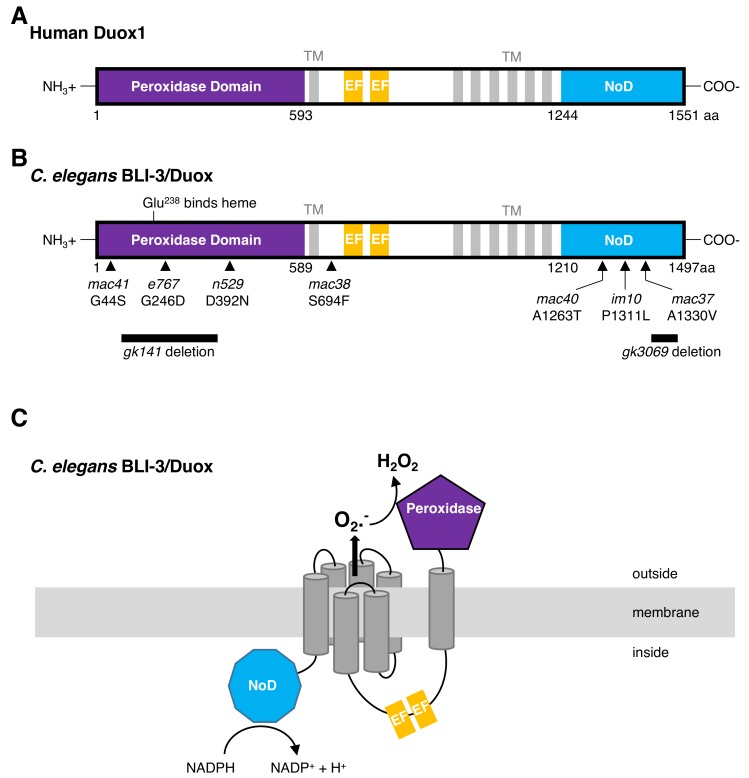
Schematic representation of human Duox1 and *C. elegans* BLI-3/Duox. (**A**) Human Duox1. (**B**) *C. elegans* BLI-3/Duox. Arrowheads and arrows indicate mutation; in italics are the allele names; and below, the amino acid substitution. (**C**) Proposed topology model of *C. elegans* BLI-3/Duox. For (**A**–**C**), TM = transmembrane region, EF = EF-hand, NoD = NADPH oxidase domain.

The nematode *C. elegans* encodes two Duox genes (*bli-3* and *duox-2*) that share a 94% identical amino acid sequence to each other, and about 30% to human Duox 1 and 2 [32]. Similar to human, the *C. elegans* Duoxs are 7-transmembrane-spanning proteins containing an intracellular flavin adenine dinucleotide (FAD)-binding and NADPH-oxidase domain, two intracellular EFhand calcium-binding domains, and an extracellular peroxidase domain (Figure 2). The amino acids involved in calcium ligation of the EF-hand calcium binding domain are poorly conserved, suggesting that the *C. elegans* Duox might not be activated by calcium binding [32]. In support of this observation, calcium was not required to stimulate ROS production when the *C. elegans* Duox was heterologously expressed in cultured human cells [36]. By contrast, the peroxidase and the putative FAD-binding domains, as well as the NADPH-binding regions, are up to 90% conserved between *C. elegans* and humans [32]. The *C. elegans* Duox BLI-3 peroxidase domain covalently binds heme (Figure 2B), a prerequisite for its catalytic function [37] and, in vitro, the peroxidase domain of BLI-3 showed similar peroxidase activity compared to the human Duox1 [32]. Furthermore, treating *C. elegans* with a flavoprotein inhibitor diphenyleneiodonium (DPI) blocked BLI-3/Duox-induced ROS production *in vivo* [13]. Together, this suggests that the *C. elegans* peroxidase and NADPH-oxidase are fully functional, and that calcium might not be required for *C. elegans* Duox activation. Hence, the current model of *C. elegans* BLI-3/Duox function is that the intracellular NADPH-oxidase domain uses NADPH to reduce oxygen to superoxide, which is then rapidly converted into hydrogen peroxide by its extracellular peroxidase domain (Figure 2C).

## 3. Tissue Distribution of Duox in *C. elegans*

The *C. elegans* Duox genes (*bli-3* and *duox-2*) are located on the same chromosome, about 2 kb apart in opposite directions, and show high similarity, suggesting a gene duplication event during evolution. Both genes are expressed at low levels, but *bli-3* expression strongly correlates with the molting cycle during development, whereas significant *duox-2* mRNA expression has not been observed [32]. Since no function of the *C. elegans duox-2* has yet been observed [38,39], this review will focus on *bli-3*, and all further mentioning of *C. elegans* Duox mainly refers to BLI-3. A note of caution, since *bli-3* and *duox-2* mRNA sequences are highly similar, RNA interference (RNAi) against *bli-3* might also knock down *duox-2*.

The BLI-3/Duox is predominantly expressed in epidermal tissue and in the gastrointestinal tract. Using BLI-3 antibodies, the BLI-3 protein has been detected on the cell surface of the *C. elegans* hypodermal cells in a “string-of-pearls” pattern [32]. A promoter of *bli-3* fused to green fluorescent protein (GFP) showed *bli-3* expression in the intestine, hypodermis, and neurons [40]. Transgenic expression of mCherry, driven by the *bli-3* promoter and including the first two exons, showed that *bli-3* is expressed in the pharynx, hypodermis, and intestine [38]. This truncated BLI-3-fused mCherry expression product localizes to the cell membrane of the hypodermis and the apical membrane of the intestine in a punctate-manner [38], reminiscent of the previously mentioned “string-of-pearls” pattern. It would be interesting to know whether these BLI-3 puncta are important for its Duox function.

## 4. The Maturation Complex of Duox in *C. elegans*

**Figure 3:**
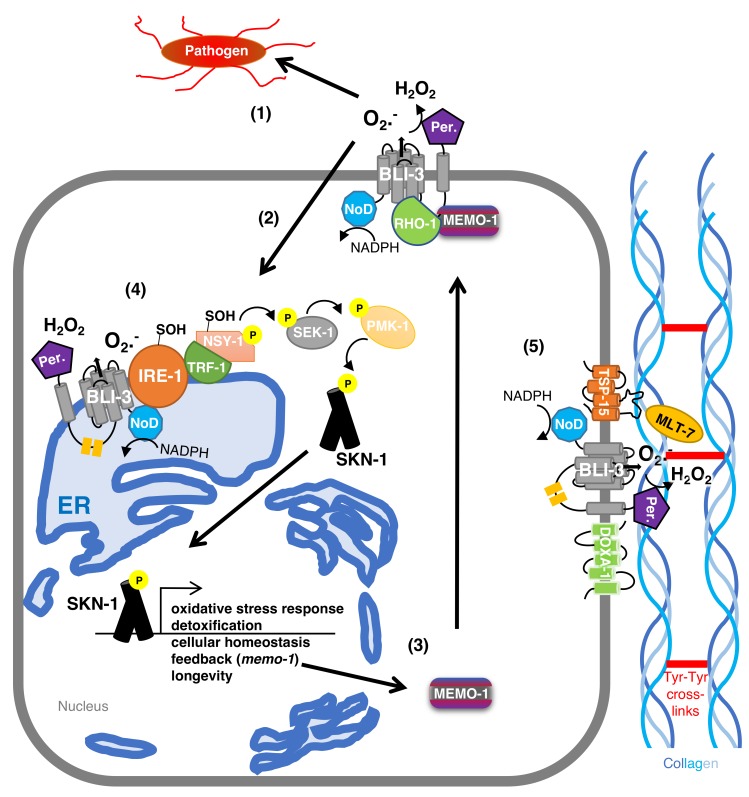
Model of BLI-3 functions This model of BLI-3 functions includes (1) ROS bursts at the cell surface to kill invading pathogens, (2) ROS as a signaling molecule from the cell surface BLI-3 to p38 MAPK signaling to SKN-1, (3) the feedback loop to MEMO-1 important for healthy aging, (4) endoplasmic reticulum localized BLI-3 to sulfenylate IRE-1 leading to TRF-1 recruitment and NSY-1 activation signaling to p38 MAPK pathway to SKN-1 orchestrating the oxidative stress response, and (5) during development BLI-3 generated ROS together with MLT-7 to crosslink collagens.

In an elegant genetic screen searching for Duox-characteristic phenotypes, mutations in *bli-3*/Duox, *doxa-1*/DOXA, and *tsp-15*/TSP have been isolated [36]. Similar to mammals, the *C. elegans* dual oxidase maturation factor DOXA-1 physically binds to BLI-3/Duox to recruit BLI-3 to the cell membrane [36]. The tetraspanin TSP-15 complexes together with BLI-3 and DOXA-1 (Figure 3), and this is required for the BLI-3 function [36]. Overexpression of BLI-3 is sufficient to increase ROS production *in vivo*, which is enhanced by overexpressing the three (BLI-3/DOXA-1/TSP-15) together in *C. elegans* [13], suggesting improved BLI-3 function of this matured BLI-3/DOXA-1/TSP-15 complex. Since mammalian tetraspanin is found enriched in membrane microdomains, which are important for cell–cell communication [41,42,43], it is tempting to speculate that the observed BLI-3 “string-of-pearls” pattern might represent such microdomains.

## 5. BLI-3-Generated Hydrogen Peroxide Catalyzes Collagen Crosslinking

**Figure 4:**
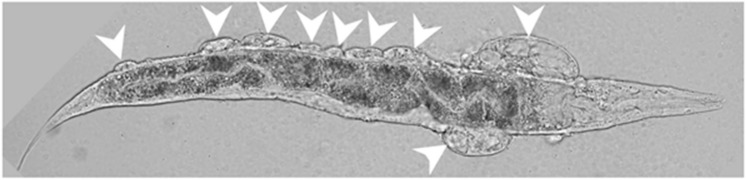
*C. elegans* showing *bli-3*-characteristic blisters. Wild type *C. elegans* (N2) was fed *bli-3* RNAi from the hatching. White chevron points to the *bli-3* blisters. Fluid material in the blisters is visible. Head to the right, ventral side down.

The Duox *bli-3* gene is an essential gene, since homozygous deletions (*gk141* or *gk3069*) or knockdown of *bli-3* by RNAi are embryonically lethal. This embryonic lethality can be rescued by expressing *bli-3* cDNA in hypodermal cells [36]. Reduction-of-function mutations cause a blistering phenotype in the developing larvae and adults, which is reflected in the gene name (*bli-3* = BLIstered cuticle-3; Figure 4). The cuticle is a collagenous extracellular matrix forming the exoskeleton of *C. elegans* [44]. Collagens are secreted from the hypodermis to integrate and form this multilayered network of the cuticle [44]. RNAi knockdown of *bli-3* solely in the hypodermis leads to a blistering phenotype [45], suggesting that BLI-3 might function at the cell surface of the hypodermis. Reducing *bli-3* function leads to detachment of the cortex layer from the basal layer of the cuticle, filling up this space with an opaque fluid (Figure 4) [32]. This fluid in the *bli-3* (mutant)-blisters is observed by electron microscopy [32], and also by GFP that is expressed in the hypodermis, but diffuses into these blisters (P*dpy-7*:GFP; [36]). These hypodermal fluid-filled blisters are not observed by other blister-causing gene mutations, such as *bli-2* and, therefore, are characteristic for *bli-3* and genes associated with *bli-3*-function, such as *tsp-15* and *doxa-1* [36]. It is unclear how the hypodermal cytoplasm leaks through the remaining basal part of the cuticle into the blisters. A likely explanation could be that the loss of cuticle integrity leads to less protection against mechanical shear forces or other insults towards the hypodermis.

The BLI-3 peroxidase domain requires heme binding to catalyze the crosslinking of free tyrosine ethyl esters to di- and tri-tyrosine linkages, as shown in *in-vitro* assays [32,46]. Around 99% of all di- and tri-tyrosine are found in the cuticle fraction, whereas these tyrosine crosslinks are almost absent in the remaining non-cuticular *C. elegans* lysates [32]. By contrast, vertebrate extracellular matrices rarely use tyrosine for collagen crosslinking, and instead use hydroxylated lysine residues for crosslinking collagens [47]. Hydroxylysine crosslinks are absent in the *C. elegans* cuticle, but found in the second extracellular matrix of *C. elegans*, the basement membrane [48,49]. *In vivo*, mutations in the peroxidase domain, as well as mutations in NADPH oxidase domain of *bli-3*, cause a blistering phenotype [36,50]. The superoxide generated by the NADPH oxidase domain is rapidly converted into hydrogen peroxide by the peroxidase domain, suggesting that mutations in the NADPH oxidase domain lead to less superoxide production required to be converted to hydrogen peroxide for the collagen crosslinking. Knocking down of *bli-3* by RNAi during development, but not during adulthood, causes this blistering phenotype [13]. Together with the observation that the expression of *bli-3* spikes during the molting cycle [32], this suggests that lowering *bli-3* function by either RNAi, mutations in the peroxidase domain, or NADPH oxidase domain, causes inadequate crosslinking of the cuticle. Since hydrogen peroxide is crucial to catalyze the tyrosine crosslinking, one would assume that complete loss-of-function mutations in the BLI-3 peroxidase domain should be lethal. Interestingly, a secreted heme peroxidase, MLT-7 (MoLTing defective), can also convert the superoxide to hydrogen peroxide in the extracellular space, thereby rescuing the function of the mutant BLI-3 peroxidase domain [36,50] (Figure 3).

An interesting twist where loss of the BLI-3 peroxidase domain might be beneficial is under magnesium-induced dopamine toxicity conditions [51]. Due to the structural similarity between tyrosine and dopamine, the BLI-3 peroxidase domain might catalyze extracellular dopamine to neurotoxic 6-hydroxydopamine leading to reduced survival when *C. elegans* is exposed to high magnesium and dopamine levels [51]. A second phenotype, where mutations in *bli-3* are protective, is under high iodide concentration [52]. Iodide in the presence of peroxidase and hydrogen peroxide can iodinate tyrosine to 3-iodotyrosine, an intermediate of thyroid hormone synthesis [53], and might also act as an inhibitor of dopamine synthesis [54]. Excess iodide leads to high ROS levels, detachment of the cuticle, and developmental arrest, which are ameliorated and bypassed by lowering BLI-3 function [52]. Therefore, to avoid collateral damage, the activity of BLI-3 must be under tight control. Taken together, during development and under normal physiological conditions, BLI-3 at the hypodermal cell surface generates superoxide, that is converted by its own peroxidase domain and by the secreted MLT-7 peroxidase to hydrogen peroxide, to catalyze tyrosine crosslinks of collagens to strengthen the cuticular extracellular matrix.

## 6. The Protective Role of BLI-3 in Pathogen Defense

In a simplistic view, the *C. elegans*’ body plan is like a tube, whereby the cuticular extracellular matrix represents the “outer” part of the tube and the cell surface of the gastrointestinal tract (mouth opening, pharynx, intestine, rectum) forms the inner part of the tube to function as physical barriers to the outside. Thereby, pathogens can either infect *C. elegans* through the cuticle or when not properly digested through the intestine. Pathogenic bacteria, such as *P. aeruginosa* [55,56], *E. faecalis* [39,55], and yeast, such as *S. cerevisiae* [57] and *C. albicans* [58], infect *C. elegans* through the intestine, whereas nematophagous fungi, such as *D. coniospora* [59,60] and *C. comatus* [60] infect through the *C. elegans*’ cuticle.

BLI-3 is required for resistance against all these above listed pathogens [39,55,56,57,58,60]. Although severe cuticle defective mutants are not susceptible to *E. faecalis* bacterial infection, suggesting the route of infection is through the intestine, both reduction of *bli-3* function, either in the hypodermis or intestine, leads to a higher mortality rate of *C. elegans* upon *E. faecalis* infection [39]. By contrast, only hypodermal reduction of *bli-3*, but not intestinal *bli-3* reduction, rendered *C. elegans* susceptible to fungal infection via the cuticle by *D. coniospora* and *C. comatus* [60].

Furthermore, all these pathogens induce BLI-3 to produce high levels of ROS [39,55,56,57,58,60], suggesting that these ROS bursts might kill off invading pathogens. For instance, *S. cerevisiae* and *C. albicans* need their own oxidative stress response to be fully functional, in order to withstand these high BLI-3-induced ROS levels and to successfully infect *C. elegans* [57,58]. Moreover, using antioxidants to neutralize the BLI-3-induced ROS upon *E. faecalis* bacterial infection diminishes the survival of *C. elegans* against this pathogen [39]. Pathogenic bacterial might be detected by released uracil, since providing exogenous uracil induced Duox-generated ROS in *C. elegans* [61]. This suggests a simple model: upon pathogen infection, BLI-3 is activated to generate high levels of ROS to eliminate these invading pathogens.

This model is only one aspect of BLI-3 function, since BLI-3 has tailored responses to different pathogen infections. Mutations in the peroxidase domain of BLI-3 (*e767* or *n529*; Figure 2B) do not lead to susceptibility of *C. elegans* towards *E. faecalis* infection [39] because another peroxidase, SKPO-1, secreted from the hypodermis, can efficiently convert the BLI-3-generated superoxide to hydrogen peroxide [62], which is analogous to the secreted peroxidase MLT-7 supporting *bli-3* function for collagen crosslinking of the cuticle. Although *skpo-1* mutants are susceptible to *E. faecalis* infection, these *skpo-1* mutants are not susceptible to *P. aeruginosa* infection [62], suggesting that either another peroxidase might be involved, or additional mechanisms confer protection against infection. For instance, upon *E. faecalis* or *P. aeruginosa* infection, the BLI-3-generated ROS activates p38 MAPK signaling to the transcription factor SKN-1(Nrf1,2,3), thereby eliciting the *C. elegans* endogenous oxidative stress response in the intestine (Figure 1, [55]). How BLI-3 is activated is unknown. Interestingly, *P. aeruginosa* infection increases proline catabolism in the mitochondria, thereby generating ROS as a byproduct released from the mitochondria that, in a presently unknown way, can stimulate the activity of BLI-3 to signal, via p38 MAPK, to activate SKN-1 to transcribe oxidative stress response genes [56]. In the hypodermis, upon fungal infection or physical injury, BLI-3-generated ROS activates CST-1/STE20 kinase, promoting nuclear translocation of the FOXO transcription factor DAF-16 to transcribe immunity genes (Figure 1, [60]). Although the downstream kinase cascade to SKN-1 and DAF-16 transcription factors and their functional importance is established, the mechanism(s) by which BLI-3 is activated upon these different infections and how BLI-3 can tailor distinct responses to these infections is not well understood.

## 7. BLI-3-Generated ROS as a Signaling Molecule to Activate the Oxidative Stress Response

ROS can modify protein function by specifically and reversibly reducing/oxidizing reactive thiol-groups on cysteine residues. If the cysteine residue is in the kinase domain, hydrogen peroxide can oxidize this thiol-group of that cysteine to the sulfenic form (Cys-SOH), causing an allosteric change that might promote or inhibit the kinase function [16]. If this kinase is in a signaling pathway, this redox change might alter the downstream biological processes. A well-established example of redox signaling is that of ROS oxidizing a cysteine residue in the protein phosphatase 1 (PTP1), thereby transiently inhibiting PTP1′s phosphatase activity [63]. For this transient redox signaling to occur, the ROS must be localized because of the high intracellular concentration of the antioxidant glutathione (about 1–10 mM in mammals [64] and *C. elegans* [65]) and, more specifically, the presence of peroxiredoxin to neutralize hydrogen peroxide [66]. In the presence of 2 mM glutathione, hydrogen peroxide potentially could travel 1.5 mm [66], the whole length of *C. elegans,* if hydrogen peroxide is actively transported across cell membranes. However, in the presence of 2 mM glutathione together with 20 μM peroxiredoxin, hydrogen peroxide can travel only for a short distance of about 5 μm [66], with a half-life time of about 1 ms [67]. These physical constrains make the ROS generated by NADPH oxidase ideal to selectively induce these redox switches to change cellular signaling pathways. In *C. elegans* and human HepG2 cells, AGC-family kinases, such as AKT, p70S6K, PKC, and ROCK1, have conserved cysteines two residues after the DFG-kinase motive, and these become sulfenylated (Cys-SOH) upon oxidative stress [34]. This sulfenylation was abolished in the presence of an NADPH-oxidase inhibitor (VAS2870) [34], suggesting the importance NADPH oxidase function for these redox switches.

Emerging and exciting evidence suggests that the ROS generated by BLI-3 acts as a signaling molecule to mediate redox signaling to promote oxidative stress resistance [13,34]. The oxidative stress response is orchestrated by the transcription factor SKN-1, which is the orthologue of mammalian Nrf1,2,3 [68]. Upon oxidative insults, a kinase cascade consisting of NSY-1/ASK mitogen-activated protein kinase kinase kinase (MAPKKK) is phosphorylated to phosphorylate SEK-1/MAPKK, which phosphorylates PMK-1/p38 MAPK, which then phosphorylates SKN-1 to allow SKN-1’s nuclear translocation and transcription of antioxidant genes (Figure 3, [68]). How NSY-1 becomes activated upon oxidative insult is unclear. However, the inorganic compound sodium arsenite is known to induce ROS and activate the p38MAPK/Nrf2 oxidative stress response in mammals and *C. elegans* [34,68,69,70,71,72]. Treating *C. elegans* with sodium arsenite leads to a physical interaction between BLI-3/Duox and endoplasmic reticulum membrane-bound kinase IRE-1 [34]. BLI-3 then generates localized ROS that sulfenylates cysteine (C663) two residues after the conserved DFG-motive in the IRE-1 kinase domain, which inhibits IRE-1 kinase activity, but leads to the recruitment of TRF-1/TRAF and NSY-1/ASK [34]. This BLI-3-IRE-1-TRF-1-NSY-1 complex leads to sulfenylation and phosphorylation of NSY-1, which then phosphorylates SEK-1 and the canonical downstream kinase cascade to SKN-1 (Figure 3) [34]. This beautiful redox signaling is essential for *C. elegans* to withstand the oxidative stress from sodium arsenite treatment and, importantly, is also well conserved in human Hep2G cells [34]. In addition, ROS generated by mitochondria can also elicit this BLI-3-IRE-1-TRF-1-NSY-1 activation complex and downstream signaling to SKN-1 [34]. Mitochondria and the endoplasmic reticulum can form connections with extensive communication between these two organelles [73]. Given the two observations that (1) bacterial pathogen infection results in mitochondrial ROS production signaling to BLI-3 to generate ROS that signals via p38 MAPK to SKN-1 [56] and (2) fungi infection to endoplasmic reticulum calcium release to activate BLI-3 to generate ROS [60], it is tempting to speculate that BLI-3 bound to the endoplasmic reticulum is activated rather than cell surface BLI-3. Furthermore, diminishing the function of these genes, *bli-3* [39,55,56,57,58,60], *ire-1* [34,74,75], *trf-1* [34,76], and *nsy-1* [77,78], leads to susceptibility to pathogen infections and to oxidative stress, whereas activating BLI-3 redox signaling to SKN-1 enables protection against pathogen infection [55] and oxidative stress [13]. This suggests that molecular insults either from chemicals, xenobiotics, or pathogens lead to the formation of this BLI-3-IRE-1-TRF-1-NSY-1 complex around the endoplasmic reticulum to initiate a redox switch to initiate the p38 MAP kinase cascade to SKN-1 to orchestrate the oxidative stress response (Figure 3).

## 8. BLI-3-Generated ROS Redox Signaling to SKN-1 to Promote Longevity

*C. elegans* that carry a mutation in either the BLI-3 peroxidase domain (*n529*) or NADPH oxidase domain (*im10*) are extremely short-lived. These two mutants show a lifespan reduction of about 20–60% compared to wild type, either on live or on heat-killed OP50 *Escherichia coli* food source [35,38]. It is worth pointing out that the standard OP50 *E. coli* used as a food source to culture *C. elegans* is mildly pathogenic during aging. Since *bli-3* is needed for protection against pathogens, the lifespan results on heat-killed OP50 *E. coli* indicate a functional role of BLI-3 during aging besides immunity. The BLI-3 peroxidase domain mutant (*e767*) has been reported to be long-lived in one study [35] and short-lived in another [38]. Since *bli-3* is important for collagen crosslinking and molting during development, the altered adult lifespan might be a consequence of developmental defects of reduced *bli-3* function. Knocking down *bli-3* specifically during adulthood, had no effect on lifespan [13]. By contrast, activating BLI-3 function either by redox co-factor pyrroloquinoline quinone (PQQ) [35], inhibiting *memo-1* [13], or by overexpressing BLI-3 [13,35] was sufficient to increase the lifespan of *C. elegans* either on alive or heat-killed OP50 *E. coli* food source. In all three circumstances, the increased lifespan was completely dependent on BLI-3-generated ROS during adulthood and on SKN-1 [13,35]. 

MEMO-1, the orthologue of mammalian mediator of ErbB2-driven cell motility, acts as a negative regulator of BLI-3 activity in *C. elegans* [13]. MEMO-1 physically interacts with RHO-1, the orthologue of mammalian small GTPase RhoA [13]. Knocking down *memo-1* frees RHO-1, leading to more RHO-1 complexing with BLI-3, and inducing BLI-3-ROS production to redox signal via the p38 MAPK pathway, to SKN-1, to transcribe oxidative stress response genes (Figure 3) [13]. Small Rho-like GTPases, such as Rac1 and Rac2, are known to activate various NADPH oxidases in mammals [19,20,79]. In *C. elegans*, BLI-3 activation and redox signaling to SKN-1 requires, in addition to RHO-1, also RAC-2 and PAK-1/p21-activated kinase [13]. Loss of *memo-1* does not result in any blister phenotype [13], suggesting that *memo-1* acts as a negative regulator for BLI-3 redox signaling to SKN-1, rather than for extracellular matrix crosslinking. Interestingly, in the promoter region of *memo-1* are two SKN-1 binding sites and overexpressing SKN-1 or constitutively activated SKN-1 leads to higher *memo-1* mRNA levels, suggesting a negative feedback loop (Figure 3) [13]. Hence, when BLI-3 activation is high, redox signaling to SKN-1 induces the expression of antioxidant and detoxification genes to neutralize the high cellular ROS levels and at the same time increases MEMO-1 levels to put the brakes on BLI-3-ROS production (Figure 1). Furthermore, BLI-3 generated ROS is important for collagen crosslinking and SKN-1 is also essential for collagen homeostasis to promote longevity [80], suggesting an intertwined interplay to strengthen the extracellular matrix integrity. In summary, these findings suggest that Duox-generated ROS acts as a redox signal via p38 MAPK to SKN-1/Nrf1,2,3 to protect against oxidative insults and re-establish cellular and organismal homeostasis to promote healthy aging in *C. elegans*.

## 9. The Role of NADPH Oxidases during Mammalian Aging

In mammals, the ROS generated by NADPH oxidases is a double-edged sword during aging. On the one hand, promoting physiological ROS generated by NADPH oxidases transiently and reversibly inhibits protein tyrosine phosphatases to potentiate insulin sensitivity and protection against high-fat-diet-induced insulin resistance in mice [81]. This suggests that transient ROS is required for fine-tuning cellular signaling to maintain normal physiology. In line with this are the observations that complete loss of NADPH oxidase function promotes age-dependent diseases and even premature aging. For instance, Nox2 knockout mice spontaneously and prematurely develop arthritis, which is increased in severity during aging [82]. Loss of Nox2 affects development and differentiation of myeloid and T cells, thereby promoting an inflammatory production and release of cytokines leading to arthritis [82]. Furthermore, loss of physiological Nox2-generated ROS accelerated the development of cellular senescence and inflammation of osteoblastic cells in the skeleton of elderly mice [83]. These findings suggest that physiological ROS generated by NADPH oxidases assist in keeping inflammatory processes under control during aging and, thus, are important for healthy aging.

On the other hand, chronic activation of NADPH oxidases has been implicated in a broad spectrum of age-dependent pathologies. For instance, during aging, the ROS from Nox1, 2, 4 contribute to molecular damage of the vascular system, potentially leading to hypertension, stroke, neuroinflammation, and dementia [84,85]. Furthermore, during aging, the Nox2 expression levels as well as Rac1 protein levels increase, leading to increased ROS levels driving cardiomyocyte hypertrophy and fibrosis in the aging hearts of rats [86]. Interestingly, these age-dependent and improper NOX overactivation phenotypes are ameliorated by exercising the rats [87]. In addition, using a NOX1-specific inhibitor to inhibit Nox1 activity in xeroderma pigmentosum type C-deficient mice rescued the accelerated aging phenotype of their skin [88]. Therefore, although chronic activation of NADPH oxidases might be a consequence of aging and might accelerate age-dependent pathologies, this chronic activation can be counteracted either via exercise or via specific Nox inhibitors.

Although NADPH oxidases are implicated in the pathogenesis of age-dependent diseases, to determine whether NADPH oxidases accelerate or slow aging, lifespan assays with altered NADPH oxidase activity are needed to be performed. For instance, increased Nox4 levels exacerbates oxidative insults of the aging heart muscles [89] and promotes cellular senescence in fibroblasts [90]. During aging, Nox4-overexpressing mice showed hypergrowth of cardiomyocytes, fibrosis, and apoptosis, presumably leading to decreased heart function [91]. Whether these Nox4-overexpression age-dependent pathologies result in a shortening of lifespan in mice has not been determined. Interestingly, increasing the activation of Nox4 by overexpression showed no detrimental health effects in young mice [91]. These young Nox4-overexpressing mice showed upregulated oxidative stress response genes [91], indicating, similar to *C. elegans* [13], an activation of NADPH oxidase to elicit the oxidative stress response as a compensatory and protective mechanism. By contrast, knockdown of Nox4 in human umbilical vein endothelial cells prolonged their cellular replicative lifespan and slowed cellular senescence [92], suggesting that reducing Nox4 activity promotes longevity in vitro. However, Nox4 knockout had no effect on mice lifespan [93]. Thus, it is questionable whether increased or decreased Nox4 activity has any effect on murine lifespan. Furthermore, these in-vivo results show that chronic activation of NADPH oxidases can be tolerated by young, but not by old animals, maybe because of the age-dependent progressive decline of protective mechanisms that normally prevent the collateral molecular damage.

## 10. Implication of NADPH Oxidases in Human Aging

In humans, mutations in genes forming the Nox2 complex (CYBB, CYBA, NCF1,2,4) cause chronic granulomatous disease (CGD), a rare inherited immunodeficiency syndrome [94]. In addition, dominant negative mutation of Rac2 was also identified in a five-week-old child with severe immunodeficiency [94]. Since CGD immunodeficient patients’ phagocytes lack Nox2 activity to generate ROS to kill pathogens, bacterial and fungal infections lead to poor survival and mostly early child deaths [95]. Thus, similar to *C. elegans*, impaired NADPH oxidase activity increases vulnerability to pathogen infections and poor survival.

By contrast, there is an association between single nucleotide polymorphism (SNP) CC for C242T in NADPH oxidase subunit (p22phox) and longevity (people >85 years) in the Turkish population [96]. In the Korean centenarian cohort, no significant difference between C242T genotype frequency was found between centenarian vs non-centenarians [97]. However, the CT and TT for C242T genotype frequency was associated with hypertension, suggesting better blood pressure for the CC genotype in elderly Korean people (>90 years) [97]. In Brazilian hypertensive patient mononuclear blood cells, the CT + TT genotype showed higher NADPH oxidase activity compared to the CC genotype of C242T p22phox gene [98], suggesting detrimental effects of improper higher ROS levels generated by NADPH oxidase. By contrast, there is an association between the A640G polymorphism in p22phox, and a reduction of systemic oxidative stress levels measured by plasma thiobarbituric acid reactive substances (TBARS) after exercise training [99], suggesting a potential redox signaling from NADPH oxidase in response to exercise training to mediate the oxidative stress response in humans. Exercise training leads to ROS formation that is important to gain health benefits from the exercise in humans [1]. Exercise is mechano-transductively linked to NADPH oxidase activity and to the activation of the Nrf2 oxidative stress response [100,101]. These findings suggest that appropriate NADPH oxidase activation via exercise might lead to redox signaling, to activate the Nrf2 oxidative stress response and to improve cellular homeostasis.

Interestingly, while studying Korean centenarians, researchers noticed a high incidence of healthy long-lived “Hansen people” [102]. This is surprising, since “Hansen people” are persons who had suffered and were cured from leprosy. The researcher noticed that these Hansen centenarians either took or are still taking 4,4′-diaminodiphenylsulfone (DDS) [102], an antibiotic commonly used to treat leprosy. DDS protects human diploid fibroblasts from oxidative insults via downregulation of NOX4 [103], and DDS is sufficient to increase *C. elegans* lifespan [104]. This further strengthens the molecular link of NADPH oxidase redox signaling to oxidative stress response mechanisms that are conserved across species.

## 11. Conclusive Remarks

ROS generated from NADPH oxidases or Duox is a double-edged sword. At physiological levels, this ROS acts on reactive thiols groups on cysteine residues (Cys-SOH) to initiate redox signaling important for the oxidative stress response and other physiological processes. During development, these physiological ROS catalyze tyrosine crosslinking of biomolecules important for extracellular matrices and tissue integrity, as exemplified by the sea urchin egg [25], *Drosophila* wing [26], the *A. gambiae* gut [27], and the *C. elegans* collagenous cuticle [32]. By contrast, high levels or bursts of ROS from NADPH oxidases are utilized to kill invading pathogens. These ROS bursts need to be neutralized via the oxidative stress response to avoid collateral damage. It makes sense that, under these ROS bursting conditions, mechanisms such as redox signaling have evolved to directly activate the oxidative stress response to detoxify, or neutralize, these free radicals, before cellular damage occurs. Upregulating these protective mechanisms makes organisms more resistant to oxidative insults. During aging, the activation or mobilization of these protective mechanisms is progressively lost. Long-lived animals are, in general, more stress resistant [105]. Selective activation of Duox during aging, upregulated the oxidative stress response genes via redox signaling to the p38 MAPK pathway to SKN-1 making *C. elegans* more oxidative stress resistant and long-lived [13]. Thus, physiological or transient ROS from NADPH oxidases acts as a signal to re-establish homeostasis, thereby promoting health during aging.
